# The Effect of Using Reactive Agility Exercises with the FITLIGHT Training System on the Speed of Visual Reaction Time and Dribbling Skill of Basketball Players

**DOI:** 10.3390/sports10110176

**Published:** 2022-11-14

**Authors:** Ahmed K. Hassan, Majed M. Alhumaid, Badry E. Hamad

**Affiliations:** 1 Department of Physical Education, College of Education, King Faisal University, Al-Ahsa 31982, Saudi Arabia; 2 Department of Team Sports and Racket Games, Faculty of Physical Education, Minia University, Minya 61519, Egypt; 3 Department of Fights and Individual Sports, Faculty of Physical Education, Minia University, Minya 61519, Egypt

**Keywords:** basketball players, dribbling skills, experimental study

## Abstract

The study aimed to determine the effect of reactive agility with the FITLIGHT training system on the speed of the visual reaction time and dribbling skill of basketball players. Participants were divided into an experimental group (n = 10; age, 14.80 ± 0.79 years; height, 163.80 ± 3.46 cm; weight, 55.90 ± 0.99 kg; training, 4.50 ± 0.53 years) and a control group (n = 10; age, 14.60 ± 0.70 years; height, 163.30 ± 3.47 cm; weight, 56.10 ± 0.74 kg; training, 4.30 ± 0.48 years). Reactive agility was assessed through a modified *t*-test, visual reaction time was assessed using the Li Lafayette instrument Visual Reaction Time Apparatus 63014 response panel, and dribbling skills were assessed through dribbling testing. The results showed improved agility through the modified agility *t*-test (11%; *p* = 0.001), an increased speed of visual reaction time for both the right and left hands (23–31%), and improved dribbling skills (19%; *p* = 0.001) for the experimental group using the FITLIGHT reactive agility software. The results also showed increased skills of the experimental group when compared to the control group. Differences in variability emerged between 6 and 14.1% in favor of the experimental group. Therefore, the researchers recommended that attention should be paid to activating the role of the FITLIGHT reactive agility training to improve and develop the physical, visual, and skill capabilities of basketball players. The researchers propose that FITLIGHT can be effectively used in the basketball training process.

## 1. Introduction

The motor components of sport skills, along with psychomotor performance, leads to success in motor performances. Factors such as anthropometric, speed, strength, endurance, coordination, and agility performance are key variables in effective athletic performance as a physical fitness index. Skill-related physical fitness consists of six parts: agility, balance, coordination, strength, speed, and reaction time [1,2]. Basketball is one of the most popular and widespread group games in most countries around the world. Many researchers [3,4,5,6,7] consider it one of the most exciting team sports for players and viewers. It is characterized by a dynamic offensive and defensive performance that requires the basketball players to possess significant agility. Agility is an essential element of team sports, including basketball. Agility is characterized by an extended start-up of body motion and a change of direction, acceleration, or rapid deceleration of physical responsiveness [8]. Abdelkrim et al. [9] indicated that agility is an important motor capability in basketball, and all offensive and defensive movements are predominantly of varied and multi-directional speeds. Pul et al. [10] showed that basketball requires agility and the ability to move quickly in different directions. Sheppard and Young [11] pointed out that the concept of agility is one of the most debated sports concepts among researchers. They claimed that there is no consensus in the sports field about the nature of agility, which may be attributed to its association with certain physical abilities [12,13,14]. Furthermore, there is a modern trend that divides agility into several components. Researchers believe that there is prior planning of closed movements performed by athletes.

Athletes know when and where to move before starting their moves to change direction. Since the situations of play are constantly and rapidly changing, another type of agility occurs. Reactive agility requires the player to quickly change direction. Re-do agility refers to when a player is put back in motion to match changing movements [15]. This could refer to rival movements, other players, the ball, or the pitch position. Surroundings are perceived by the brain through the sensory–motor receptors in the eyes, representing 70% of the total human body that can perform motor duties.

Chaalali et al. [16], Young et al. [17], and Lockie et al. [18] consider the determinants of reactive agility to be cognitive factors, including decision speed. These are performed through visual scanning, prediction, method estimation, and attitude knowledge. Physical factors, such as transitional speed, muscular torso strength, and the muscular strength properties of the two individuals, are also important. The nature of performance is also crucial. This can involve the placement of feet, the regulation of acceleration steps, and the inclination of body position. Goodman [19] adds that reactive agility is an important quality for all player positions in basketball. Game makers and wingers need agility to move past defenders when moving and changing direction. They also need it in the process of moving out of attack positions to defend and score. Agility is evident in the change in speed from forward running to a jump position, whether to aim, attack, or defend. Agility is also crucial in movements where the angles of the changes in direction are less than 90 degrees and are imposed by game situations. This qualification is also important for anchor and front-line players to be able to efficiently perform anchor and rotation. Agility is also important in the defense process, as players must perform quick defensive moves regardless of their position [20,21]. Kenneth and Bin [22] state that visual training is one of the branches of optometric science that is interested in evaluating and improving the level of visual cognition. It is a recurring series of eye-training exercises with the aim of improving the basic visual functions of sequential eye movements. It makes the player able to see the situation as a single composite unit to give him the ability to adapt to the reactive position within the overall boundaries of the pitch and the movements of the opponent. It supports good visual tracking for the purpose of improving performance skills in response to changing technical situations [23].

Researchers have argued that recently, the modern tools, devices, and means used in the training process have evolved. Their availability has become one of the reasons for success. Indeed, one of the most prominent reasons for the high level of performance among young basketball players is the use of modern tools and devices. One of the most prominent of these is the FITLIGHT technology, which is one of the devices through which effective training programs can be developed for basketball players. FITLIGHT is a modern device that is commonly used to develop some of the physical qualities and motor skills of athletes. The device is easily placed on the site or facility where training is conducted. Lights can be installed on walls, floors, tennis courts, balls, baskets, hockey pitches, and football pitches, where many different workouts and exercises can then be used. FITLIGHT is one of the devices used globally in the sports field and FITLIGHT can greatly contribute to the development of the physical, skill, and visual performance levels of young basketball players [24]. FITLIGHT improves players’ basic skills, increases performance across different sports, and develops the physical, consensual, and visual abilities of junior basketball players. This results in the production of significant responsiveness, agility, and compatibility and helps to produce a well-rounded and advanced athlete [25]. This research is a scientific attempt to recognize the impact of reactive agility training using FITLIGHT on basketball players. It also attempts to make specialists and trainers aware of the importance of training using FITLIGHT reactive agility training. Furthermore, the research highlights the significance of FITLIGHT’s role in developing players’ performance levels. The importance of training according to an individual’s abilities is also discussed. There is a need to identify the requirements of various basketball players that will allow them to use reactive agility training in accordance with their physical and skill requirements. The research proposes recommendations for researchers in different fields and sports by highlighting the importance of reactive agility exercises using FITLIGHT [11,14,22] to develop basketball players’ skills. [12,17,26]. In accordance, this study aims to clarify the effects of FITLIGHT reactive agility exercises on the visual reaction time speed and dribbling skills in basketball players. Consistently, we hypothesize that: (a) there are statistical functional differences between the pre- and post-measurement experimental group means of the reactive agility, visual reaction time (choice speed), and dribbling skills of basketball players, favoring rates of improvement after measurement; (b) there are statistical functional differences between the means of the control group before and after measurement in the reaction agility, visual reaction time velocity, and dribbling skills of basketball players, with improved rates in the post-measurement; and (c) there are statistically significant differences between the two post-measurements of the experimental and control groups in the reaction agility, visual reaction time velocity, and dribbling skills of basketball players, with the improved rates in favor of the experimental group.

## 2. Materials and Methods

### 2.1. Experimental Approach to the Problem

The study program was conducted over an eight-week period on basketball players at Hajar Club in the Eastern Province of Saudi Arabia to improve the speed of visual reaction time and the reactive skill of basketball players. Using the FITLIGHT training system, components of reactive agility training were included in the training program. The program was performed on an experimental sample of 10 basketball players with an age of 14.80 ± 0.79 years and a length, weight, and training age of 163.80 ± 3.46 cm, 55.90 ± 0.99 kg, and 4.30 ± 0.48 years, respectively. The research experiment involved a control group of 10 players who used traditional methods that did not include reactive agility using FITLIGHT exercises. The researchers supervised the training of the experimental and control groups. To identify the impact of reactive agility using FITLIGHT exercises, the results were compared between the experimental and control groups.

### 2.2. Ethical Considerations

The athletes were fully informed of the risks and benefits of the study prior to their entry and they signed an institutionally approved informed consent form. In addition, signed parental consent was obtained for the athletes. The protocol was approved by the Research Ethics Committee at King Faisal University, Saudi Arabia (KFU-REC-2021-OCT-ETHICS311).

### 2.3. Tools and Devices

**Figure 1:**
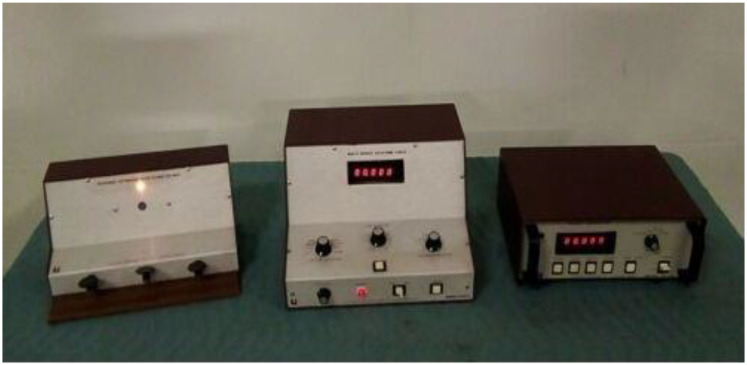
Li Lafayette Instrument Visual Reaction Time Apparatus 63014 response panel (Appendix B).

**Figure 2:**
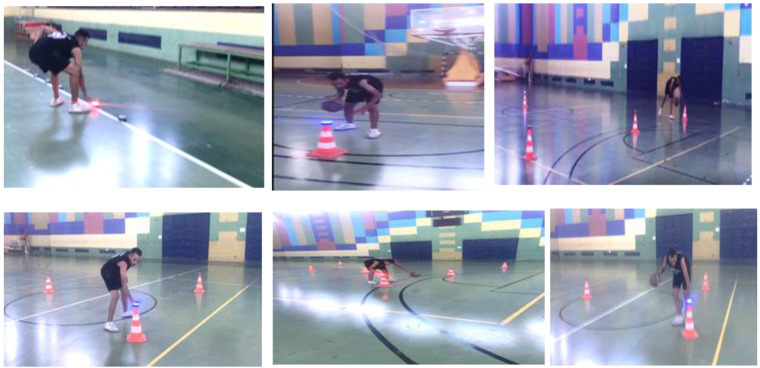
Reactive agility exercises with and without basketball (Appendix C).

A balance for weight measurements, a rastameter for height measurements, a basketball court, a basketball, colored plastic cones for use in the training program exercise app and use in a modified Agility *t*-test, a stop clock, FITLIGHT, a Visual Reaction Time Apparatus 63014 response panel for measuring visual reaction time, an agility ladder (compatibility ladder), stands for carrying the FITLIGHT disc, ropes, color adhesive tape, and a Swedish seat were used in this study. The validity and reliability of the tools and devices used were confirmed by comparing the results of the devices with other devices that measure visual interaction such as the Visual Reaction Time Apparatus 63013 response panel (Lafayette Instrument Company, 3700 Sagamore Pkwy N, Lafayette, IN 47904, USA), and the exploratory experiment was conducted by applying those measurements to another sample from outside the original sample and validating the results. Figure 1 shows a Li Lafayette Instrument (Lafayette, IN, USA) Visual Reaction Time Apparatus 63014 response panel, which contains a master control with a 1/100 s digital clock, a pre-stimulus intensity control, variable cue-stimulus delays, a stimulus selector, and a response selector; a response unit with three telegraph keys and an auxiliary; and a four-mode stimulus unit with a 1 s cue light and a three-colored stimulus light (red, green, and blue). The line voltage was 105/125 V, the AC was 50/60 Hz, the minimum reaction time was 1/100 s, the maximum reaction time was 999.99 s, the auxiliary stimulus was 6 volts, and the AC was 1 amp. Figure 2 shows the reactive agility exercises using FITLIGHT, and Table 2 shows the days, weeks, and months of the training program along with the intensity, volume, frequency, and sets (see Appendix A, Appendix B and Appendix C for more details).

### 2.4. Procedures

#### 2.4.1. Procedures to Implement Program Using FITLIGHT

The program aimed to develop the speed of visual reaction time and dribbling speed for basketball players by using FITLIGHT reactive agility exercises with or without a ball inside a stadium, simulating normal performance.

The program objectives included the following information (Table 1 and Table 2):

- The duration of the training program was 8 weeks.
- There were 4 modules per week.
- Special workouts similar to the dribbling skill performance of basketball players were selected.
- The reactive agility training began after warm-up and at the beginning of the main part of the training unit, since it placed a heavy burden on the central nervous system. This required complete preparation without fatigue.
- The reactive agility training within the training module took between 35 and 45 min.
- Breaks between workouts (1–2 s) and between groups (2–4 s) were allowed to avoid fatigue and overload.
- All players were exposed to a constant warm-up for 15 min before execution.
- The researchers took into account reactive breaks between exercises.
- The researchers used load intensity (less than maximum intensity–maximum intensity).
- The training method used over the period was high intensity.
- The number of duplicates was 2–8.
- The number of groups was 2–3.
- The rest between groups was 2–4 min.
- All exercises were performed at the highest speed possible for the individual.
- All exercises were combined with forms of play.
- Training first took place in simplified conditions and then the difficulty was gradually increased, simulating opponent time pressure.
- The diversity of exercises was as high as possible to maximize individual success experiences.

Foundations for the development of the reactive agility training program included the following:

The program was executed immediately after warming up because it required complete configuration without fatigue.

Changing and diversifying performance conditions were considered.

Changes in the implementation of motor duties and changes in the external environment were made progressively.

The workouts simulated the experience of a basketball game. 

There was progression from simple to composite exercises during the performance of reactive agility drills. 

There were links between each training exercise and between each group to avoid overload.

Exercises were first performed without a ball. After players had mastered it, the exercise was performed with the ball while performing reactive agility drills.

Reactive agility exercises were performed under accuracy and performance pressures.

The program had a series of rationed exercises for reactive agility with FITLIGHT to develop visual and skill variables for basketball players.

Table 1 shows the time distribution of the training program applied to the experimental group for a period of 8 weeks, where the program was divided into the general preparation stage, lasting 2 weeks; the special preparation stage, lasting 4 weeks; and the pre-competition stage, lasting 2 weeks.

**Table 1:** The content, objectives, and techniques used in the sports program.

Weeks	1	2	3	4	5	6	7	8
Stages	General Preparation Phase		Special Preparation Phase				Competition Preparation Phase	
Maximum			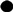			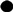	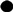	
High		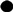		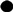				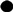
minimum	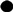				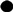			
Weekly Time	320 min	400 min	480 min	400 min	320 min	480 min	480 min	400 min
Total Time	720 min		1680 min				880 min	
Total Program Time	3280 min							

**Table 2:** The content, objectives, and techniques used in the sports program.

Stage	Weeks	Severity	Days	Warm-Up	Reactive Agility Training					Groups	Repeats	Comfort		Final
												B Exercises	B Groups	
Pre-Trial	Week 1	60%	Saturday	Exercises to prepare the body and extensions	1	2	3	4	5	2	Between 2:8 repeat, 2:3 sets according to stage and load intensity	70 s	2 min	Calming exercises
			Sunday		7	8	9	10	11	3		70 s	2 min	
			Tuesday		6	2	4	5	8	3		75 s	2 min	
			Thursday		13	14	1	2	3	3		70 s	2 min	
	Week 2	65%	Saturday		5	6	7	8	9	3		70 s	2 min	
			Sunday		11	12	13	14	1	3		80 s	2 min	
			Tuesday		6	8	9	10	11	3		80 s	2 min	
			Thursday		3	4	5	6	7	2		70 s	2 min	
Special Preparation	Week 3	70%	Saturday		9	10	11	12	13	3		75 s	2 min	
			Sunday		1	2	3	4	5	3		70 s	2 min	
			Tuesday		13	14	1	2	3	2		70 s	3 min	
			Thursday		7	8	9	10	11	3		80 s	3 min	
	Week 4	75%	Saturday		13	14	1	2	3	3		80 s	3 min	
			Sunday		5	6	7	8	9	3		75 s	3 min	
			Tuesday		15	16	17	18	19	2		70 s	3 min	
			Thursday		11	12	13	14	1	3		70 s	3 min	
	Week 5	75%	Saturday		3	4	5	6	7	3		80 s	3 min	
			Sunday		9	10	11	12	13	3		80 s	3 min	
			Tuesday		15	16	17	18	19	2		75 s	3 min	
			Thursday		1	2	3	4	5	3		70 s	3 min	
	Week 6	80%	Saturday		7	8	9	10	11	3		70 s	3 min	
			Sunday		13	14	1	2	3	3		80 s	3 min	
			Tuesday		15	16	17	18	19	2		80 s	3 min	
			Thursday		5	6	7	8	9	3		75 s	3 min	
	Week 7	80%	Saturday		11	12	13	14	1	3		70 s	3 min	
			Sunday		3	4	5	6	7	3		70 s	3 min	
			Tuesday		15	16	17	18	19	2		80 s	3 min	
			Thursday		9	10	11	12	13	3		80 s	3 min	
	Week 8	85%	Saturday		1	2	3	4	5	3		75 s	3 min	
			Sunday		15	16	17	18	19	3		70 s	3 min	
			Tuesday		13	14	1	2	3	2		70 s	3 min	
			Thursday		7	8	9	10	20	3		80 s	3 min	

Between: B.

Table 2 shows the distribution of exercises over 8 weeks and the units of the training program applied to the experimental group, as well as the time of each exercise and comfort between exercises.

#### 2.4.2. Testing Procedures

Before starting the program, a pre-measurement was conducted for all the research variables in the two groups. Firstly, there was a uniform warm-up for 15 min. This involved walking, jumping, and running short distances. All exercises were completed individually and without causing the players to become tired. Secondly, the tests were carried out in a random way to avoid fatigue from one test to another. Thirdly, verbal encouragement was given to motivate the research sample to perform each test to the best of their abilities.

The researchers performed a pre-measurement of the experimental and control research groups to test the speed of visual reaction through the use of the Li Lafayette Instrument Visual Reaction Time Apparatus 63014 response panel, the interactive agility through a modified agility *t*-test [25], and the skill of the interlocutor through a modified agility *t*-test (dribbling skill) [25], during the period of 26–27 January 2022. The researchers took into account the application of tests to all individuals in a unified manner. The proposed training program was applied for eight weeks starting on 29 January 2022 and ending on 24 March 2022 through four training units in each week on Saturday, Sunday, Tuesday, and Thursday, i.e., 32 units of the training program for interactive agility using the FITLIGHT device applied to the members of the experimental group, and the traditional program applied to the members of the control group. After the completion of the application of the program, telemetry of the two research groups was performed to test the speed of visual reaction, the interactive agility, and dribbling skill test in the period from 26 to 27 March 2022, using the same method that was followed in the pre-measurement and under the same conditions.

### 2.5. Statistical Analysis

The Statistical Package for the Social Sciences (SPSS) (IBM SPSS Statistics 26.lnk, Chicago, IL, USA) was used for the statistical analyses. The mean and standard deviation were calculated. A *t*-test analysis and the change ratio were applied in this study. The significance level was set at *p* < 0.05.

## 3. Results

**Figure 3:**
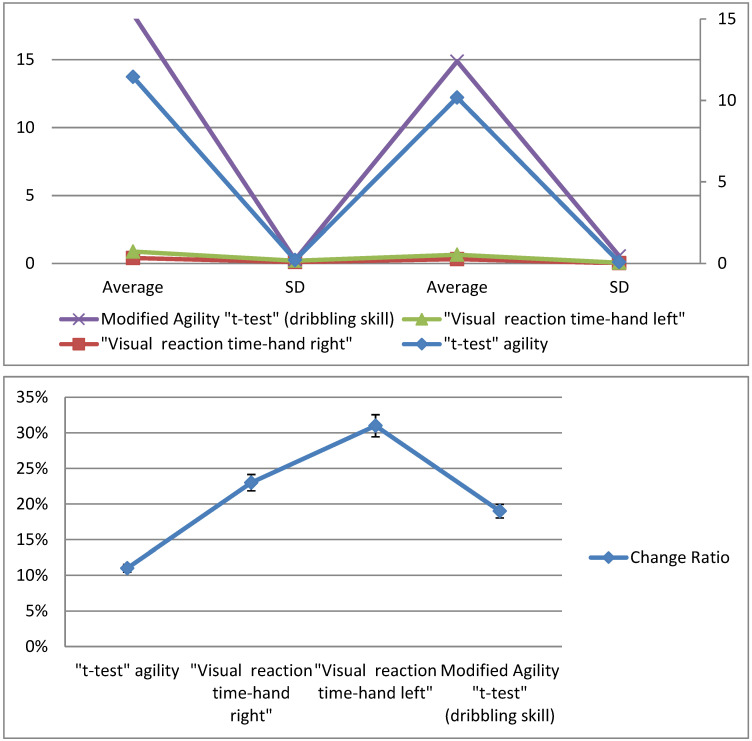
Differences between the averages of pre- and post-measurements of the experimental group for the variables under consideration.

The averages, standard deviation values, and the *t*-values of the pre- and post-measurements of the experimental group are illustrated in Figure 3 for all the variables examined for the players.

Figure 3 shows the differences between the averages of the pre- and post-measurements of the experimental group for the variables under consideration, where we found that the result of the agility *t*-test in the pre-measurement was 11.45 s, while the post-measurement was 10.18 s, with an improvement rate of 11%. In the visual reaction time test for the right hand, the pre-measurement was 0.397 ms and the post-measurement was 0.307 ms, with an improvement of 23%. The visual reaction time test for the left hand resulted in a pre-measurement of 0.476 ms and a post-measurement of 0.327 ms, with an improvement of 31%. The modified agility *t*-test (dribbling skill) resulted in a pre-measurement of 17.49 s and a post-measurement of 14.25 s, with an improvement rate of 19%. Table 3 displays the differences between the means of the pre- and post-measurements of the experimental group for the variables under consideration, the *t*-values, and the change ratios of improvement in the direction of the post-measurement.

**Figure 4:**
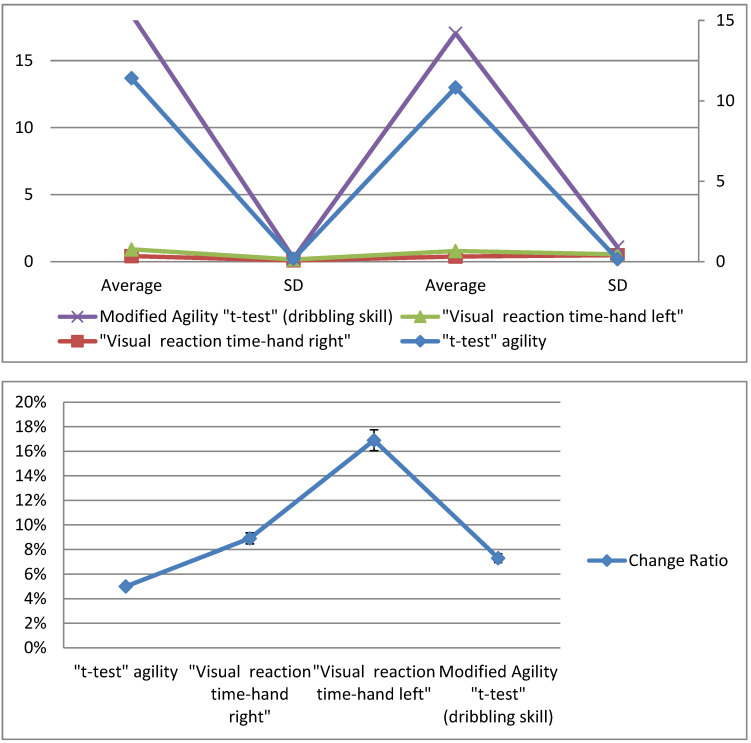
Differences between the averages of pre- and post-measurements of the control group for the variables under consideration.

Table 3 shows statistically significant differences between the two benchmarks of the experimental group for the modified agility *t*-test, the visual reaction time, and the modified agility *t*-test (dribbling skill) for the basketball players, as the calculated *t*-value was greater than the *t*-value of the tabular at an indicative level (0.05). The value of “T” in the tests in question ranged from 3.456 to 19.756, and the change ratio ranged from 11 to 31%. The averages, standard deviation values, and the *t*-values of the pre- and post-measurements of the control group are illustrated in Figure 4 for all the variables examined for the players.

Figure 4 shows the differences between the averages of the pre- and post-measurements of the control group for the variables under consideration, where we found that the result of the agility *t*-test in the pre-measurement was 11.40 s, while the post-measurement was 10.83 s, with an improvement rate of 5%. In the visual reaction time test for the right hand, the pre-measurement was 0.406 ms and the post-measurement was 0.370 ms, with an improvement of 8.9%. The visual reaction time test for the left hand resulted in a pre-measurement of 0.510 ms and a post-measurement of 0.424 ms, with an improvement of 16.9%. The modified agility *t*-test (dribbling skill) resulted in a pre-measurement of 17.52 s and a post-measurement of 16.24 s, with an improvement rate of 7.31%. Table 4 displays the differences between the means of the pre- and post-measurements of the control group for the variables under discussion, the *t*-values, and the change ratios of improvement in the direction of the post-measurement.

**Figure 5:**
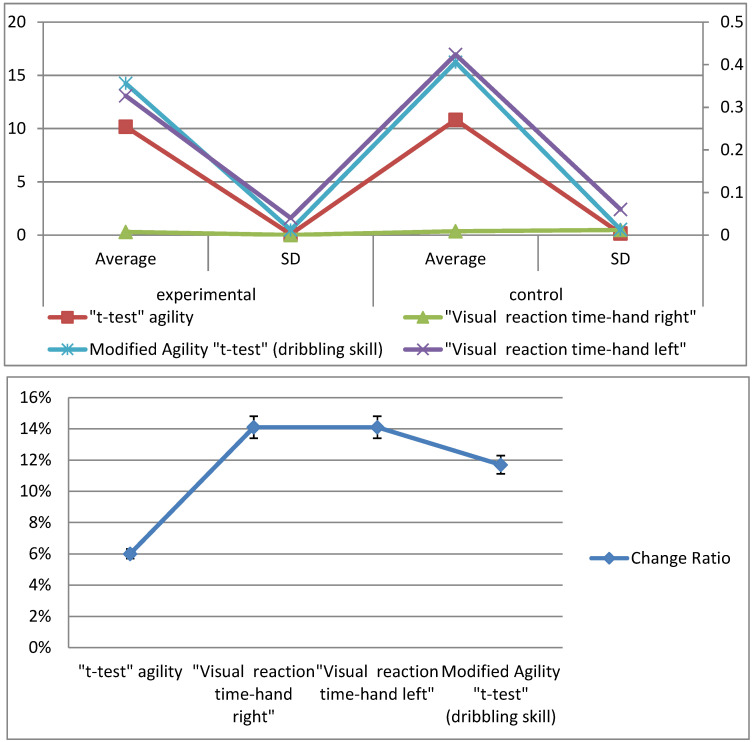
Differences between the averages of post-measurements of the experimental and control groups for the variables under consideration.

Table 4 shows statistically significant differences between the control group’s pre- and post-measurements for the modified agility *t*-test, the visual reaction time, and the modified agility *t*-test (dribbling skill) for the basketball players, as the calculated *t*-value was greater than the *t*-value of the tabular at an indicative level (0.05). The value of “T” in the tests in question ranged from 2.379 to 11.151, and the change ratio ranged from 5 to 16.9%. The averages, the standard deviation values, and the *t*-values of the two post-measurements of the experimental and control groups are illustrated in Figure 5 for all the variables examined for the players.

Figure 5 presents the differences between the averages of the post-measurements of the experimental and control groups for the variables under consideration, where we found that the result of the agility *t*-test in the experimental group was 10.18 s, while in the controlled group it was 10.83 s, with a difference in the change ratio of 6%. In the visual reaction time test for the right hand, the result for the experimental group was 0.307 ms and the result for the control group was 0.370 ms, with a difference of 14.1%. The visual reaction time test for the left hand gave a result of 0.327 ms for the experimental group and 0.424 ms for the control group, with a difference of 14.1%. The modified agility *t*-test (dribbling skill) gave a result of 14.25 s for the experimental group and 16.24 s for the control group, with a difference rate of 11.7%. As shown in Table 5, there were statistically significant differences between the experimental and control research groups for the variables under consideration. These were in favor of the experimental group, as all the calculated values of T were greater than the tabular value of T at a significance level of 0.05.

Table 5 shows statistically significant differences between the two post-measurements of the experimental and control groups for the modified agility *t*-test, the visual reaction time, and the modified agility *t*-test (dribbling skill) for the basketball players, as the calculated *t*-value was greater than the *t*-value of the tabular at an indicative level (0.05). The value of “T” in the tests in question ranged from 3.079 to 11.981, and the differences in the change ratio ranged from 6 to 14.1% in favor of the experimental group.

## 4. Discussion

The purpose of the study was to investigate the impact of FITLIGHT’s reactive agility training on the speed of visual reaction time and the dribbling skills of basketball players. The reactive agility in the modified agility *t*-test improved by 11%. Furthermore, the modified agility *t*-test for dribbling skills improved by 19% within the experimental group. The researchers attribute this result to the impact of the reactive agility training, planned and prepared in a scientific manner, that served all the physical requirements of basketball. This consisted of a series of training exercises to develop the physical and skill variables under consideration. In designing and selecting these exercises, the researchers considered the diversity, composition, and pluralism of the exercises, which gradually increased in terms of difficulty, contributing to a greater concentration of attention. Reactive agility exercises with FITLIGHT also helped the players to position their body in response to a new challenge that was suddenly presented to them during the exercise and that promoted a change of direction. This is consistent with the findings of Vencurik et al. [26], Singh et al. [27], Spasicv [28], Matthew et al. [29], and Scanlan et al. [30]. The results of these studies identified the effectiveness of reactive agility training and its positive impact on physical skills.

The improvement in the modified agility *t*-test and the modified agility *t*-test for dribbling skills can also be attributed to the effect of reactive agility using FITLIGHT exercises that are appropriate for the age group in question, and the exercises’ frequent performance with high efficiency. This is in addition to the diversity and multiplicity of these exercises, as one of the modern trends of performance development in the basketball sport to motivate players towards better performance. It was also identified that there was an improvement in the speed of visual reaction time for both the right hand and left hand of basketball players, by 23–31%. This improvement was demonstrated by the optimal use of reactive agility through FITLIGHT exercises. These exercises are linked to the theory of visual thrills, where the trainer offers players different visual thrills (lights or colors), simulating the performance that happens on the court. Indeed, reactive agility integrates dynamic perception and decision-making abilities to allow players to rapidly change direction [17,21]. This is one of the most important performance requirements of basketball. The training included various forms of variable and unstable reactions that required the player to change their place, speed, and direction of motion when performing basic skills, including dribbling skills [21,25]. Basketball players need speed to change position and perform all the game skills under time pressure. A change of direction is associated with different external stimuli, including a teammate, competitor, or ball, on the court. These depend on the decisions made by the player. These skills are improved by reactive agility drills. There was an evolution of side vision and the speed of the players’ visual reactions. There was also a clear improvement in the skill performance and in the speed of the dribbling performance of the basketball players [17,23].

Lockie et al. [18] and Millanovic et al. [31] argued that athletic performance involves optical and motor aspects. It is therefore necessary to link optical aspects using optical stimuli with performance during training, meaning that the optical aspect evolves accordingly. This study relied on reactive agility training using FITLIGHT, where physical, skill, and motor performance were linked. Reactive agility training excites the nervous system by sending lasting signals and information to the eye through photovoltaics [23]. This improved the speed of visual reaction and agility, as well as the speed of dribbling, for the basketball players.

Using reactive agility exercises with FITLIGHT excited the players, stimulated their senses, and encouraged them to exert a significant effort within the training module. The diversity and various forms of exercises added an element of suspense and limited boredom during the training, thus contributing to the development of more than one variable, whether physical, skill, or visual. Furthermore, the motor performance of the FITLIGHT exercises simulated the requirements of a basketball game and developed the motor path of physical and skill variables. These findings are consistent with the findings of both Ryan [32] and Zurek et al. [33].

The present study’s findings show an improvement in the reactive agility of the modified agility *t*-test by 5% and visual reaction time test for the right and left hands by 8.9% to 16.9%. The modified agility *t*-test for dribbling skills improved by 7.31% for the control group. The researchers attribute this improvement to the attendance of all members of the control group in training, the diversity in training, and the use of tools that help to improve agility, the speed of visual reaction time, and skill mastery. This helped each of them to break their traditional routine and repeat their skills several times during training. The traditional program contained general exercises that resulted in a high level of physical and skill performance of the players as well as continued effort. There was a significant period of exercise within the program, which resulted in the involvement of many muscle groups, thus developing muscle performance and contributing to an improvement in the speed of visual reaction time. The implementation of the training program for the control group, and its delivery of a series of graded exercises that were appropriate for the research sample’s age, had a positive impact on the tests. Therefore, the change in motor performance occurred because of regular training.

As evidenced in the present study’s findings, the experimental group outperformed the control group in the modified agility *t*-test by 6%. Within the visual reaction time tests for the right and left hands, they outperformed by 14.1%. Within the modified agility *t*-test for dribbling skills, they outperformed by 11.7%. This was due to the training program applied to the experimental group. The use of reactive agility exercises with FITLIGHT improved their vision and eye training with different orientations, which improved the speed of the players’ visual reactions. Furthermore, the FITLIGHT training improved the dribbling skills of the basketball players. The diversity of training difficulties that can be used through FITLIGHT and its richness in modern methods led to the players following the exercise instructions, and thus improving their performance. Furthermore, the training in photovoltaics contributed to an increased perception and sense of performance. This further improved the skill performance and the speed of dribbling. Reactive agility training with FITLIGHT is directly aimed at the development of physical and visual skills, which clearly improved the performance of the basketball players.

This is consistent with the findings of Delextrat et al. [12]. They argued that reactive agility exercises with visual effects contain qualitative exercises geared toward the development of certain physical and functional abilities and help to increase understanding and a sense of performance. They further help to adjust players’ movements while changing continuous external stimuli in order to simulate changing gameplay attitudes and decision-making. The use of optical stimuli hastens players’ decisions by connecting agility with dynamic perception and decision-making. Eye-sensing receptors quickly and accurately transmit external information to the brain [23], allowing rapid attitudes and timely decision-making. As 80% of the relevant information surrounding the player is transmitted through the eye, it can successfully perform the kinetic and planning duties. This is consistent with the results of Nining et al. [34], McNeil [35], Singh [27], Chaalali et al. [16], and Pauole [36]. These scholars all found that reactive agility training had a positive impact on variables in their research.

When designing the FITLIGHT reactive agility training, the researchers considered diversity and change to develop more than one physical variable and skill simultaneously. The training was characterized by diversification, excitement, and motivation. This improved motor pathways and increased motor performance, improving physical and visual skills. These exercises also involved changing the place, changing speed, changing direction, increasing strength, and encouraging compatibility. All these qualities develop physical and skill performance, and thus speed up the visual reactions of basketball players [21].

The variety of tools and devices used led to diversity within the exercises, especially FITLIGHT exercises, which is associated with improved physical and motor performance skills. This set of exercises improved the level of basic skills, which is consistent with the findings of Dawson et al. [37], Sheppard et al. [11], Farrow et al. [38], Kusnanik et al. [39], Matlák et al. [40], Humane et al. [41], and Engelbrecht et al. [42]. Reactive agility contributed to measuring and improving the speed of reaction with FITLIGHT and improving physical ability and skills. The improvement in the speed of visual reaction time was due to the impact of the training program using reactive agility with FITLIGHT exercises to raise the players’ level of performance in a rapid motor response capacity. This is because of a player’s performance of motor duties in the same motor path as the rapid initiation and implementation of short-time motor transactions of the whole body. This results from simple or composite stimuli, or could be considered a consequence of performing another motor transaction as an attitude excitement.

These differences are due to the nature of the device used to perform training. As a modern method of training, physical and skill training is characterized by fast and short movements. The differences are further due to the nature of the training used in the research, which had controlled performance times and rest periods. These periods have a great impact on the recovery of athletes, which is significant in the development of players’ performance levels. Changes in the components of the training load and the diversity of the training cause the excitement of new muscle fibers during periods of rest. This will increase the size of the muscles used in the training [43], resulting in the development of motor compatibility of the eyes, legs, and hands. The player’s skill within the exercises depended on this coordination, as the light signal forced them to move in fast and short steps, as well to use their hands to turn off the light.

## 5. Conclusions

FITLIGHT reactive agility exercises positively influenced the modified agility *t*-test. Furthermore, FITLIGHT’s reactive agility exercises positively influenced the visual reaction time test and modified *t*-test for dribbling skills. The pilot group outperformed the control group in the modified agility *t*-tests for the basketball players’ reaction times and dribbling skills. Based on the present study’s findings, several recommendations are suggested. First, FITLIGHT reactive agility training should be used when training basketball skills and visual capabilities. Second, FITLIGHT reactive agility training should be used in improving and developing the physical and skill abilities of basketball players. Third, the proposed training program using FITLIGHT exercises can be used in the basketball training process. Forth, further studies should be conducted involving programs using FITLIGHT exercises to further understand their physical, visual, and skill impacts, particularly at the junior stages. Fifth, applying the proposed training program to general sports training would allow others to benefit from its results. Finally, the principle of gradual change and diversity in training using FITLIGHT exercises can help to achieve positive results.

## Figures and Tables

**Table 3:** Differences between the means of pre- and post-measurements of the experimental group for reactive agility, speed of visual reaction time, and dribbling skill of basketball players (n = 10).

Tests	Unit of Measurement	Pre		Post		95% Confidence Interval of the Difference		T	Change Ratio	Sig
		Average	SD	Average	SD	Lower	Upper			
Agility *t*-test	s	11.45	0.21	10.18	0.08	1.086	1.460	15.395	11%	0.000
Visual reaction time—right hand	ms	0.397	0.10	0.307	0.01	0.031	0.148	3.456	23%	0.007
Visual reaction time—left hand	ms	0.476	0.09	0.327	0.04	0.101	0.196	7.058	31%	0.000
Modified agility *t*-test (dribbling skill)	s	17.49	0.08	14.25	0.49	2.867	3.610	19.756	19%	0.000

Standard deviation: SD, statistical significance: Sig, *t*-value at a significance level of 0.05 = 1.833.

**Table 4:** Differences between the means of pre- and post-measurements of the control group for reactive agility, speed of visual reaction time, and dribbling skill of basketball players (n = 10).

Tests	Unit of Measurement	Pre		Post		95% Confidence Interval of the Difference		T	Change Ratio	Sig
		Average	SD	Average	SD	Lower	Upper			
Agility *t*-test	s	11.40	0.17	10.83	0.15	0.450	0.670	11.151	5%	0.000
Visual reaction time—right hand	ms	0.406	0.09	0.370	0.48	0.002	0.070	2.379	8.9%	0.041
Visual reaction time—left hand	ms	0.510	0.07	0.424	0.06	0.060	0.112	7.494	16.9%	0.000
Modified agility *t*-test (dribbling skill)	s	17.52	0.09	16.24	0.53	0.916	1.654	7.888	7.31%	0.000

Standard deviation: SD, statistical significance: Sig, *t*-value at a significance level of 0.05 = 1.833.

**Table 5:** Differences between the two post-measurements of the experimental and control groups for reactive agility, speed of visual reaction time, and dribbling skill of basketball players (n = 20).

Tests	Unit of Measurement	Experimental N = 10		Control N = 10		T	DCR	Sig	95% Confidence Interval of the Difference	
		Average	SD	Average	SD				Lower	Upper
Agility *t*-test	s	10.18	0.08	10.83	0.15	11.981	6%	0.000	−0.763	−0.763
Visual reaction time—right hand	ms	0.307	0.01	0.370	0.48	3.079	14.1%	0.041	−0.535	−0.535
Visual reaction time—left hand	ms	0.327	0.04	0.424	0.06	4505	14.1%	0.000	−0.106	−0.106
Modified agility *t*-test (dribbling skill)	s	14.25	0.49	16.24	0.53	8.775	11.7%	0.000	−0.020	−0.020

Standard deviation: SD, statistical significance: Sig, differences in change ratio: DCR, *t*-value at a significance level of 0.05 = 2.101.

## Data Availability

The data presented in this study are available on request from the corresponding author. The data are not publicly available due to confidentiality and anonymity of the research participants.

## Appendix A. Modified Agility *t*-Test and Modified Agility *t*-Test (Dribbling Skill)

The test was conducted in two ways:

Physical incision: Performance is done without the use of the ball

The T-Test [25] is a simple running test of agility, involving forward, lateral, and backward movements, appropriate to a wide range of sports, purpose: The T test is an Reactive Agility test using Fit Light, and includes running forward, side and back, Visual reaction speed test, equipment required: tape measure, marking cones, stopwatch, timing gates (optional), Fit Light, test setup: Set out four cones as illustrated in the diagram above (5 yards = 4.57 m, 10 yards = 9.14 m), procedure: The subject starts at cone A. On the command of the timer, the person is quickly rushed to the side of the cone light by the fitlight and touches the light to be turned off with his right hand. Then he turns to the other side and alternates sideways to the lighted cone, also touching the light to turn it off, this time with his left hand. Then move aside and touch the side of the lighted cone with the right hand, and so on so that the fitlight on the B&A cone is extinguished twice, C&D once. scoring: The trial will not be counted if the subject crosses one foot in front of the other while shuffling, fails to touch Fit Light of the cones, or fails to face forward throughout the test. Take the best time of three successful trials to the nearest 0.1 seconds

Skillset: Performance is done using the ball

**Modified Agility T-Test (dribbling skill)**: The T-Test is a simple running test of agility, involving forward, lateral, and backward movements, appropriate to a wide range of sports, purpose: The T test is an Reactive Agility test using Fit Light, and includes running forward, side and back, Visual reaction speed test, To measure the speed of action of the skill of dribbling, equipment required: tape measure, marking cones, stopwatch, Basketball, timing gates (optional), Fit Light, test setup: Set out four cones as illustrated in the diagram above (5 yards = 4.57 m, 10 yards = 9.14 m), procedure: The subject starts at cone A. On the command of the timer, the person is quickly rushed with dribble to the side of the cone light by the fitlight and touches the light to be turned off with his right hand. Then he turns to the other side and alternates sideways to the lighted cone, also touching the light to turn it off, this time with his left hand. Then move aside and touch the side of the lighted cone with the right hand, and so on so that the fitlight on the B cone is extinguished twice, A&C&D once. scoring: The trial will not be counted if the subject crosses one foot in front of the other while shuffling, fails to touch Fit Light of the cones, or fails to face forward throughout the test. Take the best time of three successful trials to the nearest 0.1 seconds

## Appendix B. Visual Reaction Time Test

This accessory contains an optical reaction speed measurement device Li Lafayette Instrument Visual Reaction Time Apparatus 63014 Response Panel (Figure 1)

DESCRIPTION:The 63014, Multi-Choice Reaction Timer, This unit comes complete with a Master Control which contains a 1/100 second digital clock, pre-stimulus intensity control, variable cue-stimulus delays, a stimulus selector and a response selector; a Response Unit with three telegraph keys and auxiliary; a four mode Stimulus Unit with a 1-second cue light, a three colored stimulus light (red, green, blue), and auditory 2800 Hz Sonalert, and auxiliary; a Slide Viewer; and electronic Voice Reaction-Timer Control with desk mike, throat mike, push button box, and all the necessary cables and connectors.

SPECIFICATIONS:

Line Voltage: 105/125V AC 50/60 Hz

Minimum Reaction Time: 1/100 second

Maximum Reaction Time: 999.99 seconds

Auxiliary Stimulus: 6 volts AC to 1 amp

CONTROLS:

Power Switch: This is the main power switch of the unit and should be off while making all connections.

Initiate: The initiate switch is used to start a trial cycle, activating the cue light, stimulus and clock. Once a cycle is initiated, this key may be released; however, it does not have to be released in order for a response to be recorded. This feature is particularly useful in accurately measuring fast reaction times.

Stimulus Selector: The Stimulus Selector determines which stimulus appears with the onset of the clock. The three colored lights (blue, red, and green) and buzzer (Sonalert) are all located in the stimulus unit housing. The Viewer is used to present a 35mm slide. In the AUDIO position, the timer is initiated whenever Channel #1 of the Voice Reaction-Time Apparatus is activated. In the AUX position, any 6 volt AC device drawing up to 1 ampere will be activated. The NONE position allows the timer to be activated without any related stimulus. This may be especially useful when using the External Initiate.

Response Selector: The Response Selector switch determines which response stops the reaction timer. For example, when set to Key 1, the first telegraph key and only that will stop the timer. Any auxiliary device providing a switch closure will stop the timer when connected to the auxiliary pin sockets on the telegraph key board when the response Selector is on AUX. When in the AUDIO position, the timer will stop whenever Channel #2 of the Voice Reaction-Timer is activated.

Cue Duration: The actual cue duration is fixed at 1 second; however, this control may be used to extend the delay between cue offset and stimulus onset from 1–10 seconds. When this switch is off, the cue will not be presented.

Pre-Level Intensity: The Pre-Level intensity contact is used to keep the filament of the stimulus lamps hot to reduce thermal delay time. When this control is fully counter-clockwise, the stimulus lamps will not be visible. By adjusting this control clockwise, it is possible to perform reaction time tests on different thresholds and Jindo’s. Note that this control is only used for the three colored lamps in the stimulus unit.

Reset: The Reset push button may be used to ABORT a trial at any given time.

External Initiate: Located on the back panel, any switch closure across these binding posts will perform the same function as the front panel initiate switch.

## Appendix C

This supplement contains the reactive agility exercises using FITLIGHT (Figure 2), the days, weeks, and months of the training program, and each training session’s intensity, volume, frequency, and sets, which were selected according to the work of the muscles and the physical requirements for performing skills in basketball.
